# Vitamin B12 Protects the Exacerbated Ischemia–Reperfusion Injury-Induced Chronic Kidney Disease in Mice with Genetically Increased Elmo1

**DOI:** 10.3390/antiox14111277

**Published:** 2025-10-24

**Authors:** Jiayi Zhou, Yuye Wang, John Hagaman, Qing Ma, J. Charles Jennette, Meitong Chen, Xianwen Yi, Yukako Kayashima, Nobuyo Maeda-Smithies, Feng Li

**Affiliations:** 1 Department of Nutrition, Gillings School of Global Public Health, The University of North Carolina, Chapel Hill, NC 27599, USA; jz0105@email.unc.edu; 2 Department of Pathology and Laboratory Medicine, School of Medicine, The University of North Carolina, Chapel Hill, NC 27599, USA; yuye_wang@med.unc.edu (Y.W.); hage@med.unc.edu (J.H.); maqing@email.unc.edu (Q.M.); charles_jennette@med.unc.edu (J.C.J.); cmeitong@email.unc.edu (M.C.); xianwen_yi@med.unc.edu (X.Y.); yukaya@email.unc.edu (Y.K.); nobuyo@med.unc.edu (N.M.-S.)

**Keywords:** acute kidney injury, chronic kidney disease, Ischemia–reperfusion injury, oxidative stress, ELMO1, vitamin B12

## Abstract

Ischemia–reperfusion injury (IRI) is a leading cause of acute kidney injury (AKI) and a major driver of progression to chronic kidney disease (CKD). Oxidative stress is recognized as a central mediator of this transition. Engulfment and Cell Motility 1 (ELMO1) regulates cytoskeletal remodeling and reactive oxygen species generation through Rac1 activation, but its contribution to CKD progression remains poorly defined. To investigate this, we established a unilateral renal IRI model in wild-type (WT) and *Elmo1*-overexpressing (*Elmo1^H/H^*) mice and evaluated kidney function one and four months post-IRI. Compared with WT, *Elmo1^H/H^* mice developed more severe kidney dysfunction, including an elevated plasma cystatin C and urinary albumin-to-creatinine ratio, reduced estimated glomerular filtration rate (eGFR), and pronounced fibrosis and glomerular injury observed by light and electron microscopy. Molecular analysis confirmed the dysregulation of redox-related pathways by RT-qPCR, with RNA sequencing showing enrichment of oxidative stress signatures. A subset of mice received chronic vitamin B12 (B12) supplementation following IRI to evaluate its therapeutic potential. Vitamin B12 supplementation improved kidney function, reduced fibrosis, preserved glomerular structure, and normalized the expression of antioxidant genes in both groups. These findings identify *Elmo1* as a driver of redox-mediated kidney injury and support vitamin B12 as a promising antioxidant therapy for AKI-to-CKD progression.

## 1. Introduction

CKD affects approximately 14% of adults in the United States and is more prevalent among older adults, women, and non-Hispanic black individuals according to the 2022 Epidemiology Chronic Kidney Disease Report [1]. CKD can progress to end-stage kidney disease (ESKD) and significantly increases the risk of cardiovascular morbidity and mortality [2]. Ischemia–reperfusion injury (IRI) is a common cause of AKI, a major risk factor for CKD, in both clinical and experimental settings [3,4]. The progression from AKI to CKD involves maladaptive repair, including persistent inflammation, tubular atrophy, capillary loss, and fibrosis, altogether leading to irreversible renal failure [5]. No effective therapies exist to prevent or reverse IRI-induced AKI or halt CKD progression [6]. Evidence also shows systemic effects, with contralateral kidney injury driven by circulating inflammatory/oxidative factors, indicating AKI impacts whole-body homeostasis [5,7].

Genetic predispositions, including single nucleotide polymorphisms (SNPs) in genes in oxidative stress regulation and immune signaling, have been linked to modulate susceptibility to AKI and CKD [8]. Notably, SNPs in the *Engulfment and Cell Motility 1* (*ELMO1*) gene have been associated with kidney diseases such as diabetic nephropathy and nephrotic syndrome [9,10]. Our previous work showed that physiological variation in *Elmo1* (20–200%) links diabetic complications via increased reactive oxygen species (ROS) production [11,12]. Elmo1 activates Rac family small GTPase 1 (Rac1) through dedicator of cytokinesis (Dock) proteins, promoting NADPH oxidase-dependent ROS generation. Oxidative stress is a key mediator of IRI-induced kidney injury and AKI-to-CKD progression [13].

In this study, we examined the role of high *Elmo1* expression in kidney damage progression at one month and four months after unilateral IRI, using wild-type (WT) and *Elmo1^H/H^* mice, which express approximately twice the WT level of *Elmo1* mRNA [11]. We showed that *Elmo1* overexpression worsened CKD-like phenotypes in both affected and contralateral kidneys, with altered redox genes, heightened immune response, and increased fibrosis, indicating that overexpressed *Elmo1* amplifies injury beyond the primary site. Because vitamin B12 (cobalamin) has potent antioxidant properties and our prior study demonstrated its protective effects against IRI [14], we tested whether vitamin B12 could execute any beneficial effects on AKI-induced CKD progression in this context. Our findings highlight redox imbalance as a key driver in disease progression and support vitamin B12 as a readily available candidate for therapeutic intervention, particularly during the early transition phase.

## 2. Methods

Mice: The *Elmo1^H/+^* allele originally generated by Hathaway et al. [11] was placed on a C57BL/6J background through a series of backcrossing for more than 21 generations. WT and *Elmo1^H/H^* littermates from *Elmo1^H/+^* pairs in both sexes at the age of 12–16 weeks were housed in standard cages on a 12 h light/dark cycle and were allowed free access to food and water. All experiments were carried out in accordance with the National Institute of Health’s guidelines for the use and care of experimental animals, as approved by the IACUC of the University of North Carolina at Chapel Hill (protocol number #: 25-190).

Renal ischemia/reperfusion (IR) procedure: Mice were anesthetized with 1.5% isoflurane inhalation, and core body temperature was maintained at 37 °C. A midline laparotomy was performed; the left renal pedicle was clamped using microaneurysm clamps for 30 min to induce ischemia. The clamps were then removed to allow reperfusion, and the incision was closed. In sham-operated controls, the renal pedicle was left untouched, but the abdomen remained open for 30 min with exposure of the left renal pedicle as previously described [14].

Vitamin B12 treatment: Mice with IR surgery were randomly enrolled into either vehicle (water) or B12 treatment groups at a dose of 50 mg/L via drinking water immediately after surgery for four months. Rodent chow (Cat# 3002909–203, PicoLab, Fort Worth, TX, USA) contained B12 at 79 μg/kg [14].

Biochemical analysis: One and four months after surgery, spot urine was collected before euthanasia. Blood and tissues were collected after euthanasia. Plasma cystatin C, Endothelin-1 (ET-1), corticosterone, and urinary albumin were measured by ELISA kits. (MSCTC0, DET100, R&D Systems, Inc., Minneapolis, MN, USA; 80556, Crystal Chem, Elk Grove Village, IL, USA; Albuwell M kit #1011, Ethos Biosciences, Logan Township, NJ, USA). Mouse urine creatinine was measured by an enzymatic assay kit (80350, Crystal Chem, Elk Grove Village, IL, USA).

Morphological examination: After perfusion with 0.1% heparin in phosphate-buffered saline (PBS), tissues were fixed with 4% paraformaldehyde. Fixed tissues were embedded in paraffin and sectioned (5 μm), then stained with Masson’s trichrome and hematoxylin and eosin staining (H&E) [15,16]. Semi-quantification of tubular injury was performed by an investigator who was blinded to the experimental groups. Evaluation of tubular loss was adapted from the Interstitial Fibrosis and Tubular Atrophy (IFTA) method from the Banff working classification of renal allograft pathology, but counting the actual number of glomerulus numbers per field [17]. For each kidney section, five fields per slide were captured under a standard field of view using light microscopy (Olympus BX43, Tokyo, Japan). The standardized field size was estimated using scale-calibrated images on imaging software. Images were captured with cellSENS Microscope Imaging Software (version 4.3, Evident Olympus, Tokyo, Japan).

Blood Pressure Measurement: Systolic blood pressure was measured using a non-invasive tail-cuff photoplethysmography machine (Visitech BP-2000) (Visitech Systems Inc. Apex, NC, USA) at one month and four months post-surgery [18].

Transmission Electron Microscopy (TEM): Tissues were collected and fixed in 2% paraformaldehyde/2.5% glutaraldehyde in 0.1 M sodium phosphate buffer, pH 7.4 for 1 h at room temperature and stored at 4 °C until processing. Approximately three sections (2 mm) of cortex were dissected out and were washed with 0.15 M sodium phosphate buffer (pH 7.4) three times for 5 min each, then incubated in 1% osmium tetroxide in 0.1 M sodium phosphate buffer for 1 h at room temperature. Ultrathin sections (80 nm) were cut using a diamond knife on a Leica UCT7 ultramicrotome (Leica Miscrosystems, Wetzlar, Germany) and applied to 200 mesh copper grids. Grids were stained with 4% aqueous uranyl acetate for 12 min followed by Reynold’s lead citrate for 8 min (Reynolds, 1963) [19]. Samples were viewed using a JEOL 1400 Flash transmission electron microscope operating at 80 kV (JEOL USA, Inc., Peabody, MA, USA) and images were acquired with an AMT NanoSprint61 ActiveVu: High Resolution CMOS TEM camera (Advanced Microscopy Techniques, Woburn, MA, USA).

Quantitative Reverse Transcription Polymerase Chain Reaction (qRT-PCR): Total RNA from tissues was extracted using TRIzol^®^ (Invitrogen, ThermoFisher Scientific, Waltham, MA, USA) following the manufacturer’s instructions. mRNA was quantified with TaqMan qRT-PCR (QuantStudio 3 real-time PCR systems, ThermoFisher Scientific, Foster City, CA) by using the one-step qRT-PCR Kit (Bio Rad, Hercules, CA, USA) with *18s* as the reference gene in each reaction. The 2^−ΔΔCt^ method was used for comparing the data. The sequences of primers and probes used are listed in Table S1.

RNA Sequencing: Total kidney RNA was extracted using RNeasy mini kit (Qiagen, Hilden, Germany) according to the manufacturer’s protocol. Library preparation and sequencing were performed by High Throughput Sequencing Facility (HTSF) at UNC-Chapel Hill. Paired-end raw sequences of 100 bp were acquired by NextSeq 2000 (Illumina, San Diego, CA, USA), and subsequent demultiplexing was performed using bclconvert with a 1-mismatch threshold to segregate reads based on indexes. Raw sequencing reads were preprocessed using FASTP to remove adapters and trim poly-G tails. The trimmed reads were then aligned to the Ensemble gene database (release 113) using the SALMON aligner. The gene count matrices were generated and extracted from the SALMON quantification files. The raw counts were then filtered and normalized for the subsequent differential gene expression analysis using relevant Bioconductor packages in R studios (Posit Software, PBC Version 2024.12.1+563).

Statistical analysis: The data are presented as mean ± standard error of the mean (SEM) unless otherwise stated. A multifactorial analysis of variance test was used with the program JMP^®^ Pro 17.2.0 (SAS Institute Inc., Cary, NC, USA). Group differences were analyzed using one-way or two-way analysis of variance (ANOVA). For one-way ANOVA, pairwise comparisons were conducted using Student’s *t*-tests. A *p*-value < 0.05 was considered statistically significant.

## 3. Results

### 3.1. Elmo1^H/H^ Mice Had Severely Compromised Kidney Function than WT Counterparts Four Months After IRI

**Figure 1:**
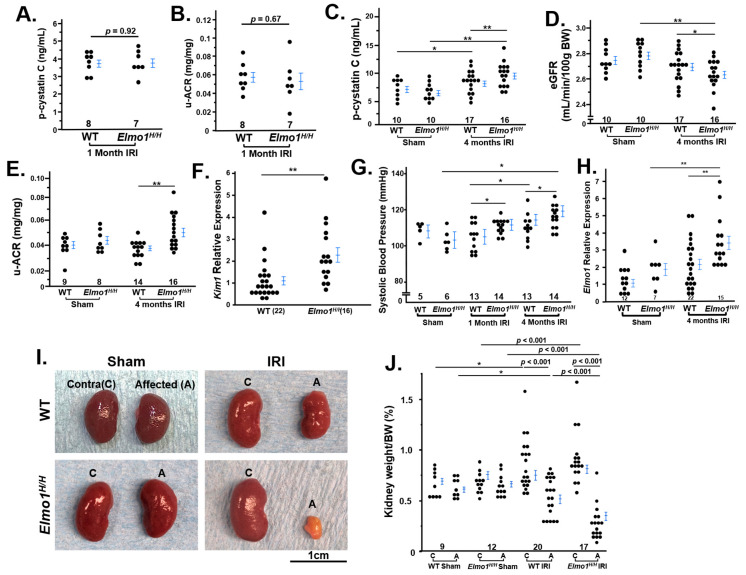
*Elmo1^H/H^* mice show greater kidney dysfunction and renal mass changes compared with wildtype (WT) 4 months after unilateral ischemia–reperfusion injury (IRI). (**A**) Plasma cystatin C (p-cystatin C) levels and (**B**) urine albumin-to-creatinine ratio (u-ACR) levels were not different between the *Elmo1^H/H^* and WT IRI group at one month after surgery. (**C**) The p-cystatin C level and (**D**) u-ACR was significantly higher in the *Elmo1^H/H^* IRI group at four months after surgery. (**E**) Estimated glomerular filtration rate (eGFR) showed it was significantly decrease in the *Elmo1^H/H^* IRI group at four months after surgery. (**F**) *Kim1* mRNA level in the contralateral kidneys is significantly upregulated in the *Elmo1^H/H^* IRI group. (**G**) Systolic blood pressure at different time points after surgery. (**H**) *Elmo1^H/H^* mice exhibit approximately a 2-fold increase in *Elmo1* expression compared to WT mice, and IRI further increased *Elmo1* expression after four months. (**I**) Comparison of kidney size in both WT and *Elmo1^H/H^* mice from sham and IRI groups. (**J**) Kidney weights were normalized to body weight (BW). * *p* < 0.05, ** *p* < 0.01, *p* values smaller than 0.001 are listed as *p* < 0.001. # (represents numbers) of animals are indicated in the figures.

We evaluated overall renal injury and dysfunction by measuring plasma cystatin C, the urinary albumin-to-creatinine ratio (u-ACR), and estimated glomerular filtration rate (eGFR) as complementary indicators of kidney function [20]. One month after IRI, we observed no significant difference in the kidney functions between *Elmo1^H/H^* and WT groups (Figure 1A,B). Four months after IRI, plasma cystatin C levels increased in WT and *Elmo1^H/H^* mice, to a significantly greater extent in *Elmo1^H/H^* mice (Figure 1C). eGFR decreased in *Elmo1^H/H^* mice (Figure 1D) while these mice had higher u-ACR (Figure 1E). Kidney injury molecule 1 (Kim1) is a transmembrane damage marker associated with inflammation and fibrosis [21,22]. It is encoded by *Kim1* gene, and its expression was more than twice as high as the expression in contralateral kidneys of *Elmo1^H/H^* mice compared with WT (Figure 1F), demonstrating that increased *Elmo1* accelerates IRI-induced renal damage and dysfunction in mice during the CKD progression.

As kidneys regulate hormones and electrolytes [23], we observed an earlier and greater rise in systolic blood pressure (SBP) in *Elmo1^H/H^* mice after one month IRI and remained elevated throughout, while WT showed a later rise; by four months both groups exceeded sham controls and *Elmo1^H/H^* mice still had higher SBP than WT counterparts (Figure 1G). *Elmo1^H/H^* mice had a 2x higher mRNA level of *Elmo1* compared with WT as expected (Figure 1H). Endothelin 1 (ET-1) plays an important role in AKI-CKD progression [24]. IRI mice in both groups had higher plasma ET-1 than their sham counterparts and surprisingly, *Elmo1^H/H^* mice at four months had significantly lower ET-1 than in WT after IRI, (Figure S1A). Plasma corticosterone levels did not show a statistic difference among the four groups of mice (Figure S1B).

Four months after IRI, both WT and *Elmo1^H/H^* mice showed a loss of renal mass in the IRI kidney, with greater atrophy in *Elmo1^H/H^* (Figure 1I). About 25% of *Elmo1^H/H^* IRI kidneys appeared pale, suggesting poor circulation and/or fibrosis [25]. Contralateral kidneys remained at approximately 1% of body weight and showed compensatory enlargement (Figure 1J). When evaluating the relationship between contralateral kidney size and affected kidney size, we noticed that sham mice showed little variation between the two kidneys (Figure S2A). Compensatory growth of the contralateral kidneys post-IRI appeared to be limited in the WT mice and two thirds of the *Elmo1^H/H^* mice (Figure S2B). Three out of 14 *Elmo1^H/H^* mice had further growth compensation of the contralateral kidneys. The affected kidneys of those mice had severe atrophy (0.2% of body weight). These findings indicate that *Elmo1* overexpression worsens injury while enhancing compensatory remodeling of the uninjured kidney.

There was no difference between WT and *Elmo1^H/H^* mice who underwent sham surgery (Figure 1).

### 3.2. Elmo1^H/H^ Mice Had More Severe CKD-like Morphological Changes, Especially in the Affected Kidneys

**Figure 2:**
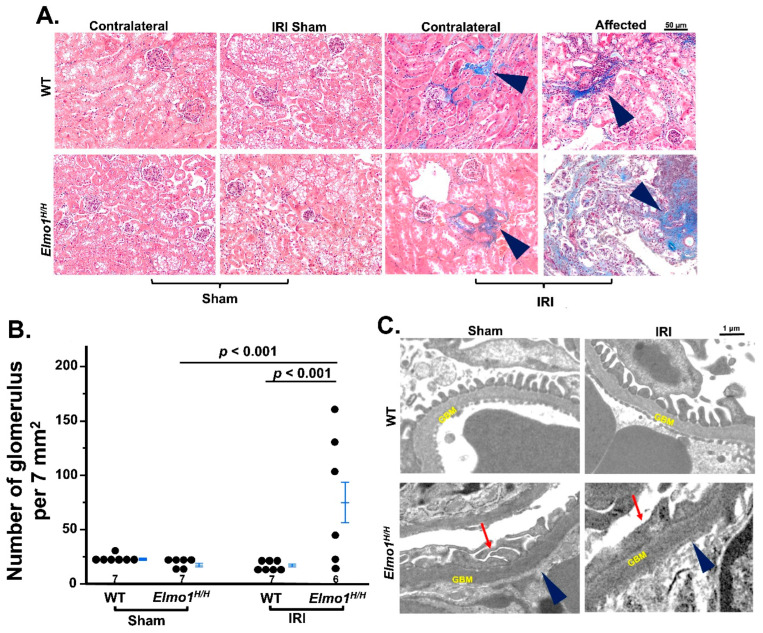
*Elmo1^H/H^* mice with unilateral ischemia–reperfusion injury (IRI) show severely impaired kidney structures compared to wild-type (WT) mice. (**A**) Representative Masson’s trichrome staining of kidney sections from sham and IRI groups four months after surgery (20×). Histology figures illustrated are among the most severely damaged areas from each group. Arrowhead: fibrosis. (**B**) Semi-quantification of the number of glomeruli per standardized field of view (4×). (**C**) Transmission electron microscopy (TEM) of affected kidneys highlights ultrastructural damage, including podocyte foot process effacement (red arrow), and loss of endothelial fenestration (blue arrow) (5000×). GBM: glomerular basement membrane. Note: foot process and endothelial fenestration are intact in WT mice. *p* values smaller than 0.001 are listed as *p* < 0.001.

Next, we evaluated the histological changes in kidneys at four months after surgery. Under light microscopy, there were no remarkable changes at one month after surgery (Figure S3). After four months, the evaluation of fibrosis by Masson’s trichrome staining showed that *Elmo1^H/H^* mice had more severe tubulointerstitial fibrosis and glomerulosclerosis in both IRI and contralateral kidneys than WT counterparts (Figure 2A) [26]. Tubular atrophy and loss were seen in affected kidneys in *Elmo1^H/H^* mice, as evidenced by clusters of glomeruli, reflected in a higher glomerular number per field (Figure 2A,B). Transmission electron microscopy (TEM) examination in the affected kidneys showed loss of endothelial fenestration, podocyte foot effacement, and loss of slits in glomeruli, which were not present in WT counterparts [27,28] (Figure 2C). Sham kidneys did not show any obvious abnormalities in WT and *Elmo1^H/H^* mice under light microscope and TEM (Figure 2A,C).

### 3.3. Elmo1^H/H^ Mouse Kidneys Showed Redox Imbalance Four Months After IR Surgery

**Figure 3:**
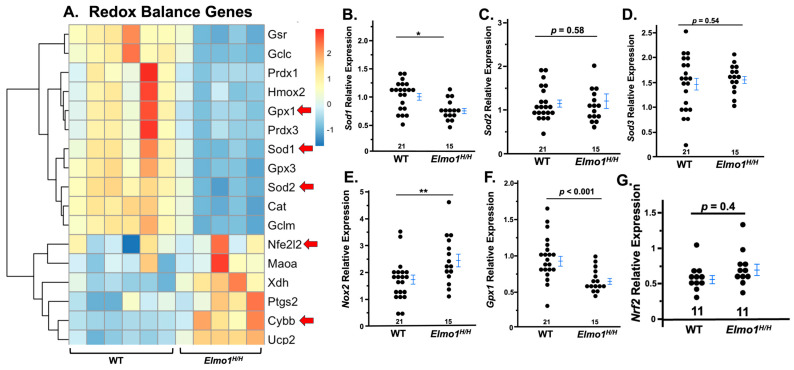
Whole-kidney RNA sequencing on the affected kidneys (**A**) and rt-qPCR of redox genes on contralateral kidneys four months after ischemia–reperfusion injury (IRI) (**B**–**F**). (**A**) Heatmap of selected redox balance-related genes qRT-PCR was performed to assess oxidative stress, red arrows indicate all the genes for which RT-PCR was performed; (**F**,**G**) gene expression in the contralateral kidneys of WT and *Elmo1^H/H^* mice. * *p* < 0.05, ** *p* < 0.01, *p* values smaller than 0.001 are listed as *p* < 0.001.

To investigate the molecular basis of kidney function four months after IRI, we analyzed RNA expression patterns by RNA sequencing (RNA-Seq). We used six WT and five *Elmo1^H/H^*-affected kidneys. The samples were selected as their affected kidney weight relative to their contralateral kidney weight fell within the average range (0.62 ± 0.057% in 20 WT, 0.44 ± 0.062% in 17 Elmo1H/H). ROS plays an important role in CKD progression and severity [29], and Elmo1 overexpression is linked to increased ROS in diabetic cardiomyocytes [12]. Therefore, we assessed the expression of genes related to the redox system four months after surgery. RNA-seq analysis of affected kidneys revealed a significant disruption of redox balance between WT and *Elmo1^H/H^* groups, with *Elmo1^H/H^* mice showing impaired antioxidant responses (*Gpx1*, *Gpx3*, *Cat*) and an upregulation of pro-oxidant genes (*Ptgs2*, *Cybb*, *Ucp2*) in *Elmo1^H/H^* kidneys, potentially contributing to tissue injury and fibrogenesis as shown under histological examination (Figure 3A).

Superoxide dismutase 1 (Sod1) is a key cytosolic enzyme encoded by *Sod1* that converts superoxide radicals into hydrogen peroxide. Its gene expression was markedly reduced in contralateral kidneys from *Elmo1^H/H^* mice with IRI compared to WT counterparts (Figure 3B), while no differences in *Sod2* or *Sod3* were observed (Figure 3C,D). *Glutathione peroxidase 1* (*Gpx1*), an essential antioxidant enzyme that detoxifies hydrogen peroxide to water, was also downregulated in *Elmo1^H/H^* mice with IRI (Figure 3E). *NADPH oxidase 2* (*Nox2*), a major pro-oxidant gene, increased ~2.5-fold (Figure 3F), indicating redox imbalance in the contralateral kidney, and confirmed *Elmo1*′s role in enhancing ROS production.

Among all cellular defense pathways, *Nrf2* works by binding antioxidant response elements (AREs) in DNA, which activates many genes involved in antioxidant defense [29]. In the *Elmo1^H/H^* mice, *Nrf2* did not exhibit significant change compared to the WT counterparts (Figure 3G), which indicate the global antioxidant effects might go through other defense mechanisms. On the ischemic side, *Nfe2l2*, which encodes the transcription factor of Nrf2, showed a mixed pattern between both genotypes (Figure 3A).

Because oxidative stress is a key driver of tissue remodeling and chronic progression in kidney injuries [30,31], we next examined whether redox imbalance in *Elmo1^H/H^* mice was accompanied by changes in fibrotic and inflammatory pathways. Heatmaps showed a consistent trend of upregulated gene profiles in *Elmo1^H/H^* mice, with an increased expression of *Tgf-β*, *Col1a1*, *Col4a1*, *Fn1*, and *Vim*, consistent with extracellular matrix deposition [32], tubular dedifferentiation, and maladaptive remodeling (Figure S4). Fibrosis- and inflammation-related gene expressions in contralateral kidneys largely mirrored changes in IRI kidneys, indicating a systemic response. Although many changes were not statistically significant, the pattern suggests that fibrosis extends beyond the injured kidney. Inflammatory genes displayed a similar trend, with *Il6* and *Tnfα* elevated, *Tlr4* suppressed [33], and acute chemokines *Ccl2* and *Cxcl1* reduced, consistent with the chronic-phase suppression of immune trafficking [34] (Figure S5).

Furthermore, we examined transcriptional changes related to DNA damage, as excessive ROS can induce both genomic and mitochondrial injury [35,36]. In ischemic kidneys from both genotypes, *Elmo1^H/H^* mice showed marked upregulation of *Lig1*, *Brca1*, and *Chek1*, indicating enhanced DNA repair activity and damage response, consistent with more severe cellular injury (Figure S6A). We also observed that all the genes important for mitochondrial metabolism and energy processing were downregulated in the affected kidneys (Figure S6B). this reflects severe damage of mitochondria in the proximal tubular cells in the IRI-affected *Elmo1^H/H^* kidneys occupied with zebra bodies [37], (Figure S6C). Many of these genes are closely linked to nutrient and fatty acid metabolism. This coordinated suppression aligns with the observed tubular atrophy and loss of function, suggesting mitochondrial dysfunction, metabolic dysfunction, and impaired capacity for ROS detoxification (Figure S6B).

These findings parallel the redox imbalance observed in affected kidneys, as sustained oxidative stress can amplify fibrotic and inflammatory signaling pathways, exacerbating DNA damage and metabolic reprogramming. Together, the data indicate that *Elmo1*-driven ROS accumulation exacerbates local injury and promotes inter-kidney crosstalk, leading to systemic oxidative stress-linked fibrosis and maladaptive remodeling.

### 3.4. Vitamin B12 (B12) Markedly Improved the Kidney Function and Structure Four Months After IR Surgery

**Figure 4:**
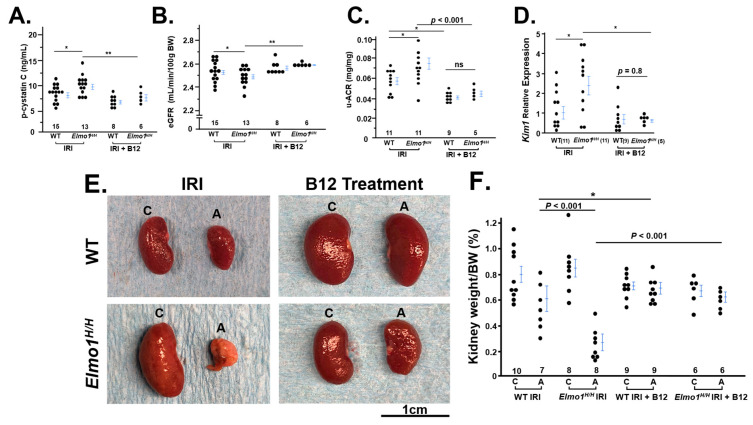
Vitamin B12 (B12) treatment improves renal function and preserved renal mass in *Elmo1^H/H^* mice with ischemia–reperfusion injury (IRI). (**A**) Plasma cystatin C (p-cystatin C) levels were reduced in both WT and *Elmo1^H/H^* IRI groups following B12 treatment, indicating improved kidney function. (**B**) u-ACR was significantly decreased in both genotypes after B12 supplementation. (**C**) eGFR was improved in B12-treated *Elmo1^H/H^* mice compared to untreated controls, supporting the renoprotective effect of B12. (**D**) *Kim1* mRNA level was significantly reduced in the B12 treated group. (**E**,**F**) Kidney size and weight were corrected by B12 treatment in both groups. * *p* < 0.05, ** *p* < 0.01, *p* values smaller than 0.001 are listed as *p* < 0.001. Two-way ANOVA analysis is presented in Supplemental Table S2.

Since B12 is a potent antioxidant, and we have previously demonstrated that B12 acute supplementation decreases renal superoxide and post-IRI in mice [14,38], we evaluate whether B12 protects the severe phenotypes of CKD noted in *Elmo1^H/H^* mice. Following four months of vitamin B12 treatment, both WT and *Elmo1^H/H^* mice exhibited improved kidney function relative to untreated IR groups. Plasma cystatin C levels and u-ACR in B12-treated mice were closer to sham levels, indicating preserved glomerular filtration and reduced kidney damage, along with the restoration of eGFR in both injury groups (Figure 4A–C). *Kim1* mRNA level was also significantly lower in the B12 treated group compared to the IRI group (Figure 4D). B12 treatment preserved renal mass and kidney weight in both genotypes (Figure 4E,F).

**Figure 5:**
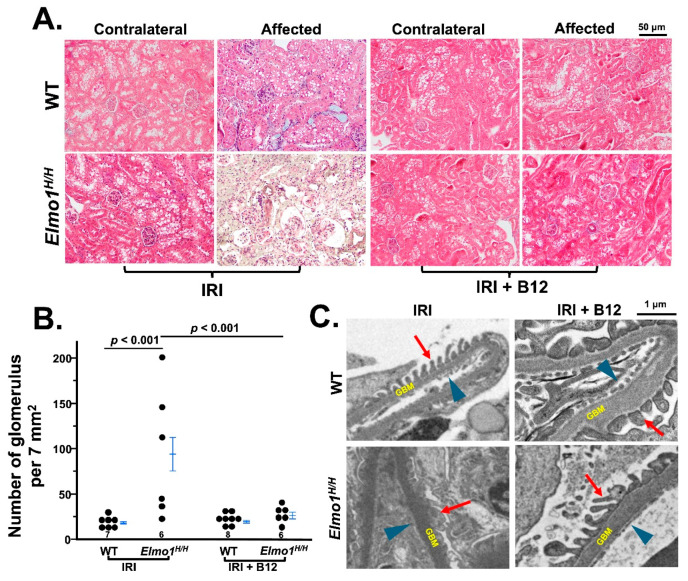
Vitamin B12 preserves renal structures in IRI mice, reducing fibrosis and glomerular damage. (**A**) Representative Masson’s trichrome staining of kidney sections shows that vitamin B12 treatment reduces interstitial fibrosis and glomerular damage in both WT and *Elmo1^H/H^* IRI mice compared to vehicle-treated groups. (**B**) Semi-quantification of glomeruli per field of view demonstrates reduced glomerular density in B12-treated *Elmo1^H/H^* mice, indicating the preservation of tubular architecture. (**C**) Transmission electron microscopy of affected kidneys reveals that B12 treatment preserves foot process structure, and endothelial fenestration in both genotypes. Blue arrowhead: endothelial fenestration. Red arrow: foot process. GBM: glomerular basement membrane. *p* values smaller than 0.001 are listed as *p* < 0.001. Two-way ANOVA analysis is presented in Supplemental Table S2.

Chronic vitamin B12 treatment also significantly attenuated kidney structural and morphological injury following IRI, as demonstrated by both light and electron microscopy. Masson’s trichrome staining revealed a marked reduction in interstitial and vascular fibrosis in B12-treated mice (Figure 5A), as indicated by a lower glomerular count per field of view compared to vehicle-treated counterparts (Figure 5B), reflecting the preservation of tubular and interstitial spaces and notable preservation of glomerular architecture in both WT and *Elmo1^H/H^* IRI groups. Histological evaluation also revealed reduced tubular atrophy and restored cortical perfusion, suggesting improved oxygen delivery to the lateral kidney border (Figure 5A). On the ultrastructural level, electron microscopy confirmed that B12 treatment preserved podocyte foot processes, endothelial fenestration, and the glomerular basement membrane structure, reversing the extensive cellular damage observed in vehicle-treated *Elmo1^H/H^* mice (Figure 5C).

**Figure 6:**
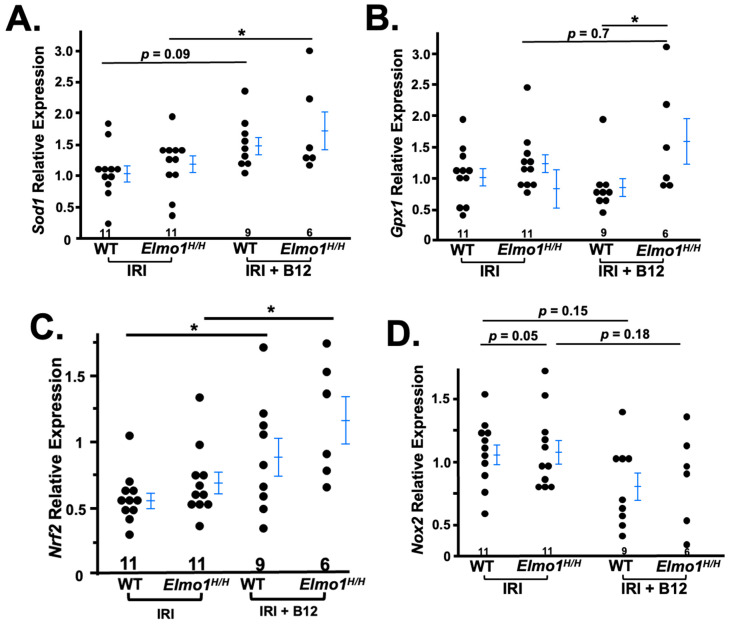
Vitamin B12 corrects the expression of genes related to redox after four months of B12 treatment. The expression levels of *Sod1*, *Nrf2*, *Nox2*, and *Gpx1* are shown as fold changes relative to the WT-IRI group. Both vitamin B12-treated groups exhibited a restoration expression of antioxidant genes (**A**–**C**) and a decreased expression of the pro-oxidant gene *Nox2* (**C**). Pro-oxidant gene *Nox2* showed downregulated after B12 treatment (**D**). * *p* < 0.05. Two-way ANOVA analysis is presented in Supplemental Table S2.

We further compared gene expression profiles between treated and untreated groups. We specifically analyzed the expression of key oxidative stress-related genes involved in antioxidant defense. In B12-treated mice, the expression of antioxidant genes *Sod1*, *Nrf2,* and *Gpx1* was significantly increased (Figure 6A,B,D), indicating restoration of redox homeostasis. Additionally, *Nox2* expression was reduced, suggesting decreased oxidative stress during the adaptive repair phase (Figure 6C) [39]. These results support the role of vitamin B12 in enhancing antioxidant capacity and mitigating ROS-mediated damage in the context of IRI.

Collectively, the data suggest that the beneficial effects of B12 are mediated through its antioxidant function, which likely contribute to the attenuation of chronic kidney disease progression.

### 3.5. Female Mice Developed Less Severe CKD-like Phenotypes After IRI than Males Four Months After IRI, While Effects of High Elmo1 Persisted

**Figure 7:**
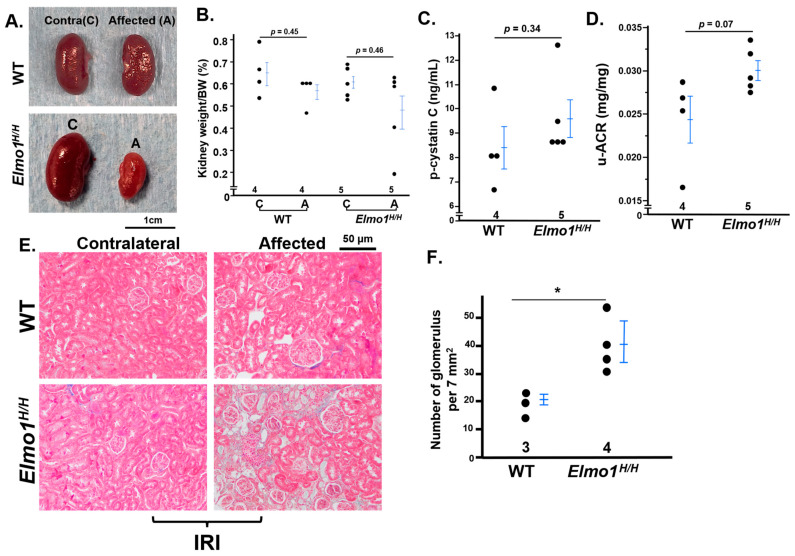
Female *Elmo1^H/H^* mice following kidney ischemia–reperfusion injury (IRI) exhibit less severe CKD-like phenotypes compared to males after four months. Compared to male counterparts, *Elmo1^H/H^* female mice showed less severe CKD-like phenotypes after IRI. (**A**) *Elmo1^H/H^* female mice showed a decrease in kidney mass. (**B**) Kidney weight normalized to body weight (BW). (**C**) *p*-cystatin C and (**D**) u-ACR showed no significant increase compared to wild-type. (**E**) Masson’s trichrome staining of contralateral and affected kidneys, as *Elmo1^H/H^* female mice showed a low degree of fibrosis on the affected side. (**F**) Glomerular count per field of view was high in *Elmo1^H/H^* female mice, indicating tubular atrophy. * *p* < 0.05.

Although CKD is more prevalent in women [1], female mice developed only mild CKD-like phenotypes at four months after with the same IR procedure applied, less severe than males. The *Elmo1^H/H^* females showed moderate increased plasma cystatin C and u-ACR, slight kidney weight loss, and mild fibrosis (only one mouse out of six mice) (Figure 7A–E). Notably, glomerular density was higher in *Elmo1^H/H^* females than in WT females, consistent with interstitial tubular loss observed in the male counterparts (Figure 7F). These observations align with reports of sex-specific differences in AKI-to-CKD progression in mice [40], but the overexpression of *Elmo1*-enhanced CKD progression persisted in females as in males.

## 4. Discussion

In the current study, we demonstrated that *Elmo1* overexpression significantly exacerbates the progression of AKI (induced by IRI) to CKD progression. While the damage at one month after IRI was not obviously different histologically and physiologically, four months after IR surgery, *Elmo1^H/H^* male mice developed more advanced CKD-like phenotypes than WT counterparts. They showed higher plasma cystatin C and u-ACR, and drastic interstitial tubular loss, fibrosis, and glomerular damages. Additionally, antioxidant gene expression was downregulated, while genes involved in superoxide production were upregulated in *Elmo1^H/H^* mice, implicating oxidative stress as a key contributor to the aggravated CKD-like phenotype. Consistent with this, we showed that B12 (a potent SOD mimetic) supplementation immediately after surgery showed protection against the progression from AKI to CKD in *Elmo1^H/H^* mice.

The association between *Elmo1* gene variants and diabetic nephropathy was first reported by Shimazaki et al. Later, Hassan et al. showed that ELMO1 polymorphism (rs741301) is associated with nephrotic syndrome [9,10]. However, associations with other kidney diseases remain unclear. Here, we demonstrated that mice over-expressing *Elmo1* exhibited severe CKD-like phenotypes which progressed from AKI caused by IRI, providing strong evidence for its involvement in broader kidney pathologies. Interestingly, we did not observe overt kidney impairment in either WT or *Elmo1^H/H^* sham-operated mice, suggesting that elevated *Elmo1* levels alone are not sufficient to induce kidney dysfunction, but rather aggravate the progression and severity of disease under stress conditions.

In our study, both male and female *Elmo1^H/H^* mice showed greater renal functional decline and morphological damage compared with WT controls. Following 30 min of ischemia, both sexes developed renal injury, but males exhibited more severe damage, indicating that sex influences CKD progression regardless of genotype. Among *Elmo1*-overexpressing mice, females subjected to the same ischemic duration displayed milder injury, consistent with prior studies showing that females generally require longer ischemia (45–60 min) to match male injury levels [41]. While sexual dimorphism in kidney injury is largely linked to sex hormones [42], our data suggest that, under *Elmo1* overexpression, female hormones may confer partial protection and highlight potential sex-specific mechanisms in CKD susceptibility. We can further test with longer ischemic time to validate the severity of IRI in *Elmo1^H/H^* females.

Elmo1 signaling is known to regulate actin-based cytoskeletal dynamics and cell motility, and has also been linked to the activation of NADPH oxidases (Nox), which generate reactive ROS [12,43]. Our previous work confirmed the validity of the Rac1–NADPH oxidase pathway in ELMO1-driven ROS production, as oral inhibition of Rac (EHT1864) or Nox4 each partially mitigated cardiomyopathy, while pan-NADPH oxidase inhibition (VAS3947) markedly prevented it. These findings establish Rac-dependent and -independent NADPH oxidases as key mediators of ELMO1-induced oxidative stress [12]. However, because our analysis was based on whole-kidney transcriptomic data, it may not fully capture the spatial or cell-specific effects of Rac1 activation and ROS production, representing an important limitation of this study [44]. In the current study, the increased/decreased expression of antioxidant/superoxide-producing genes were observed in both affected and contralateral kidneys in *Elmo1^H/H^* mice, suggesting a systematic role for ROS in the pathogenesis in *Elmo1^H/H^* mice. To test whether CKD progression in this model is through the ROS-related pathway, we treated mice with IR surgery with B12 because of its potent anti-ROS function. Notably, chronic B12 treatment appeared to attenuate the progression from AKI to CKD, suggesting that oxidative stress plays a key role in *Elmo1*-driven renal pathology. However, excessive ROS generation may trigger a cascade of detrimental effects beyond oxidative stress, including DNA damage, mitochondrial dysfunction, and metabolic reprogramming due to impaired nutrient handling [45]. These changes can further lead to cell cycle dysregulation and the activation of cell death pathways [46], collectively exacerbating tissue injury and accelerating the AKI-to-CKD transition.

B12 (cobalamin) is the largest vitamin, containing a central cobalt atom within a corrin ring [47]. Although synthesized only by certain bacteria and archaea, cobalamins are essential coenzymes for nearly all life (except plants); in vertebrates, methylcobalamin and adenosylcobalamin are required for methionine synthase and methylmalonyl-CoA mutase [48]. Once inside the cell, the oxidation state of the cobalt atom is reduced from Co(III) to Co(II) and to Co(I). A prior study has reported that Co(II)balamin is a highly effective intracellular superoxide (O_2_^•−^) scavenger with a reaction rate close to that of superoxide dismutase (SOD) [49]. Our previous work showed that B12 mitigates IRI-induced AKI by reducing oxidative stress, inflammation, fibrosis, DNA damage response, and apoptosis [14]. In addition, B12 plays a crucial role in the maintenance of one-carbon metabolism, enhances S-adenosylmethionine (SAMe) production and methylation capacity [50]. In Akita diabetic mice, B12 restored reduced *Dnmt1/3a/3b* expression, increasing the methylation of *Socs1* and *Socs3* promoters [50]. These results suggest that B12 mitigates diabetes-induced epigenetic dysregulation. Future work will assess B12-dependent enzymes, methionine synthase (MTR) and methylmalonyl-CoA mutase (MUT), to clarify its role in one-carbon metabolism and mitochondrial function in CKD [51]. It is highly likely that B12 could exert protective effects through mechanisms beyond superoxide scavenging [52].

In this study, a single high dose of vitamin B12 administered immediately after IR surgery effectively prevented CKD progression in both WT and *Elmo1^H/H^* mice without detectable adverse effects. However, in patients with renal impairment, accumulation of toxic B12 metabolites such as thiocyanate may limit therapeutic benefit [53]. Our previous work demonstrated that much lower doses (approximately one-tenth of the current dose) were sufficient to prevent diabetic cardiomyopathy in mice [54], indicating that both dose and timing are critical determinants of efficacy and safety. Additional studies from our laboratory tested B12 at 1, 10, and 100 mg/kg/day, with 50 mg/L corresponding to roughly 10 mg/kg/day, and even the highest dose showed no evident toxicity. Extending treatment into the chronic phase may further preserve kidney function, as prior findings demonstrated that B12 reduced acute kidney injury even when administered up to five days post-IRI [14]. Previous interventions also showed that genetic signaling pathway inhibitors and nephrectomy have limited success in slowing kidney disease progression [55,56]. Since B12 treatment began immediately after injury and continued throughout the study, future work should refine dose and timing to maximize protection while minimizing toxicity. Further studies must determine whether effects are specific to cyanocobalamin, as thiocyanate accumulation from impaired metabolism or external sources may worsen oxidative stress and vascular injury in CKD. Evaluating safer alternatives like methylcobalamin could better define effective and translational B12-based strategies.

This study has several limitations. First, the findings are based primarily on a single mouse strain (C57BL/6J) with *Elmo1* overexpression, which may limit generalizability across genetic backgrounds. Second, although our results identify key oxidative stress–related pathways, the study lacks deeper mechanistic exploration to delineate how vitamin B12 modulates redox signaling, mitochondrial metabolism, and gene regulation. Additional work is needed to clarify whether the observed effects are specific to the cyanate structure of B12, as differences in bioavailability and metabolism may influence both efficacy and potential toxicity. Third, while B12 supplementation showed protective effects, the high experimental dose and lack of long-term toxicity evaluation warrant caution in clinical translation. Lastly, the complexity of human CKD, including systemic oxidative stress that may alter inter-organ responses, storage, and metabolic balance—further limits the direct extrapolation of our findings. Future studies should employ multi-organ analyses and mechanistic models to strengthen biological insight and translational relevance.

## 5. Conclusions

Our study demonstrates that *Elmo1* overexpression accelerates AKI-to-CKD progression by amplifying oxidative stress, fibrosis, and inflammation while reducing compensatory capacity in the contralateral kidney. This highlights *Elmo1* as a critical mediator of kidney–kidney crosstalk and a potential therapeutic target. Mechanistically, *Elmo1*-driven Rac1–NADPH oxidase activation likely enhances ROS generation, sustaining a pro-fibrotic microenvironment. Early, high-dose vitamin B12 supplementation effectively halted CKD progression in *Elmo1^H/H^* mice, consistent with its antioxidant and cytoprotective properties. Together, these findings underscore *Elmo1*′s pathogenic role in kidney injury and support the translational potential of B12, warranting further studies to optimize timing, dosing, and long-term safety in preventing CKD progression.

## Data Availability

RNA sequencing data are deposited to the NCBI Gene Expression Omnibus (accession: GSE304219).

## Supplementary Materials

The following supporting information can be downloaded at https://www.mdpi.com/article/10.3390/antiox14111277/s1, Supplemental Method [57]; Table S1. qRT-PCR primers and probes list; Table S2. Two-way ANOVA Analysis; Figure S1. Plasma endothelin-1 (ET-1) (A) and plasma corticosterone levels (B) across experimental groups; Figure S2. Correlation between the affected (IRI kidney) and contralateral kidney (Contra) weights of each animal with sham (A) and IRI (B); Figure S3. Kidney histological changes in wildtype and *Elmo1^H/H^* male mice one month after IRI; Figure S4. Fibrosis gene expression shown by RNA-seq heatmap (A) in affected kidneys of WT (*n* = 6) and *Elmo1^H/H^* (*n* = 5) mice and by qRT-PCR (B–F) in contralateral kidneys; Figure S5. Inflammatory gene expression shown by RNA-seq heatmap (A) in affected kidneys of WT (*n* = 6) and *Elmo1^H/H^* (*n* = 5) mice and by qRT-PCR (B–F) in contralateral kidneys; Figure S6. DNA damage and repair related genes and mitochondrial related gene expression shown by RNA-seq heatmap (A,B) in affected kidneys of WT (*n* = 6) and *Elmo1^H/H^* (*n* = 5) mice. (C) TEM of affected kidneys showing ceramides/lipids containing lysosomes (zebra bodies) in the proximal tubular cells.
