# Supplementary material for: Vitamin B12 Protects the Exacerbated Ischemia–Reperfusion Injury-Induced Chronic Kidney Disease in Mice with Genetically Increased Elmo1
Source: Antioxidants (Basel). 2025 Oct 24;14(11):1277. doi: 10.3390/antiox14111277 (PMC12649674; doi:10.3390/antiox14111277)
Supplement: Supplementary file 1 [file antioxidants-14-01277-s001.zip › antioxidants-3905656-supplementary.pdf]

Supplemental Materials

Vitamin B12 protects the exacerbated ischemia-reperfusion injury induced chronic-kidney-disease in mice with genetically increased Elmo1

Jiayi Zhou[1,2], Yuye Wang[2], John Hagaman[2], Qing Ma[2], J. Charles Jennette[2], Meitong Chen[2], Xianwen Yi [2], Yukako Kayashima[2], Nobuyo Maeda-Smithies[2], and Feng Li[2 *]

[1]Department of Nutrition, Gillings School of Global Public Health, The University of North Carolina, Chapel Hill, NC 27599, USA.

[2]Department of Pathology and Laboratory Medicine, School of Medicine, The University of North Carolina, Chapel Hill, NC 27599, USA.

*Correspondence to: Feng Li Ph.D., Department of Pathology and Laboratory Medicine, The University of North Carolina, Chapel Hill, NC 27599, USA.

Phone: 919-597-0864. Fax: 919-966-8800.

E-mail: lif@med.unc.edu

Method: Estimated glomerular filtration rate (eGFR) calculation: Plasma cystatin C (p-cystatin C) levels by ELISA and calculating eGFR values using the following formula: eGFR (mL/min/100g body weight) = -3.2886 * p-cystatin C (μg/mL) + 2.9409[1].

Table S1. qRT-PCR primers and probes list

Gene		Sequence (5’-3’)
Ccl2	Forward	CTG GAG CAT CCA CGT GTT G
ID: 20296	Reverse	TGG GAT CAT CTT GCT GGT GA
	Probe	FAM-AG CCA GAT GCA GTT AAC GCC CCA CT—TAMRA
Col1a1	Forward	AGA GCA TGA CCG ATG GAT TC
ID: 12842	Reverse	ATT AGG CGC AGG AAG GTC AG
	Probe	FAM-CT CCG ACC CCG CCG ATG TCG —TAMRA
Col4a1	Forward	GGC TAT TCC TTC GTG ATG CA
ID: 12826	Reverse	CTA AAC TCT TCC AGA CAG GAC
	Probe	AGG TTC CGG CCA AGC CCT CGC
Cxcl1	Forward	GCT TGT AGG TGT TGC CCT C
ID: 14825	Reverse	AGG CAA GCC TCG CGA CCA T
	Probe	FAM-AC CCA AAC CGA AGT CAT AGC CAC ACT C—TAMRA
Fibronectin	Forward	CAA GTC GAT GCC ATC CCA G
ID: 14268	Reverse	CTG GCT GTA AAC CTG TAA TGG
	Probe	FAM-AT GGC CAG ACC CCA GTT CAG AGG A—TAMRA

Gpx1	Forward	GTC TGG GAC CTC GTG GAC
ID: 14775	Reverse	TTC TTG CCA TTC TCC TGG TG
	Probe	FAM-TG GTG CTC GGT TTC CCG TGC AAT CAG—TAMRA
Il6	Forward	CTC TCT GCA AGA GAC TTC CA
ID: 16193	Reverse	CTC TCC GGA CTT GTG AAG TA
	Probe	FAM-CTG ATG CTG GTG ACA ACC ACG GCC T—TAMRA
Kim1	Forward	CAA GTT AAA CCA GAG ATT CCC AC
ID: 171283	Reverse	CGT GAT GCT GAG AAG TCT CA
	Probe	FAM-CT GTC CAT CTC TGG TCT CAA CCG TC—TAMRA
Nfe2l2	Forward	CAT GAG TCG CTT GCC CTG GA
ID: 18024	Reverse	ATT GAG GGA CTG GGC CTG AT
	Probe	FMA-TC CCC AGC CAC GTC GAA AGT TCA G— TAMRA
Sod1	*Forward*	*CCA TTG AAG ATC GTG TGA TCT C*
ID: 20655	Reverse	CTT GTT TCT CAT GGA CCA CC
	Probe	FAM-CA GGA GAG CAT TCC ATC ATT GGC CG—TAMRA
Sod2	Forward	AAG GAA CAA GGT CGC TTA CA
ID: 20656	Reverse	AGC AGC GGA ATA AGG CCT GT

	Probe	FAM-TG CTG CCT GCT CTA ATC AGG ACC CA—TAMRA
Sod3	Forward	CCT TCT TGT TCT ACG GCT TG
ID: 20657	Reverse	CTG GAC TCC CCT GGA TTT GA
	Probe	FAM-TG ACA GAG CCA CAG GCC GCC AGT—TAMRA
Tgfβ1	Forward	TGC TTC AGC TCC ACA GAG AA
ID: 21803	Reverse	GTG GAT CCA CTT CCA ACC CA
	Probe	FAM-CC TTC CTA AAG TCA ATG TAC AGC TGC CG-TAMRA
Tlr4	Forward	GGT GAG AAA TGA GCT GGT AAA G
ID: 21898	Reverse	GCA ATG GCT ACA CCA GGA AT
	Probe	FAM-TG CCC CGC TTT CAC CTC TGC CTT CA—TAMRA
Tnfα	Forward	CAC ACT CAG ATC ATC TTC TCA A
ID: 21926	Reverse	AGC TGC TCC TCC ACT TGG T
	Probe	FAM-AG CCT GTA GCC CAC GTC GTA GCA--TAMRA
Vim	Forward	CTG GTT GAC ACC CAC TCA AA
ID: 22352	Reverse	CGT GAT GCT GAG AAG TCT CA
	Probe	FAM-CT GTC CAT CTC TGG TCT CAA CCG TC—TAMRA
18s	Forward	AGA AAC GGC TAC CAC ATC CA

ID: 19791	Reverse	CTC GAA AGA GTC CTG TAT TGT
	Probe	FAM-AG G CAG CA G GCG CGC AAA TTA C—TAMRA

Elmo1 TaqMan assay is purchased from ThermoFisher Scientific (Waltham, MA) with the lot number Mm00519109_m1. (ID: 140580)

Table S2. Two-way ANOVA analysis

Genotype	Treatment	p-cystatin C (ug/mL)	u-ACR (mg/mg)	eGFR (mL/min/ 100gBW)	Contra/BW (%)	Affected/ BW (%)	# of glomerular	*Kim1* mRNA	*Sod1* mRNA	*Gpx1* mRNA	*Nox2* mRNA	Nrf2 mRNA
WT IRI	No B12	9.2 ± 0.8	0.03 ± 0.003	3.3 ± 0.2	8.0 ± 0.4	5.4 ± 0.4	17 ± 10	1.3 ± 0.3	1.1 ± 0.1	1.1 ± 0.3	1.3 ± 0.3	0.6 ± 0.1
Elmo1$^{H/H}$ IRI	No B12	10.6 ± 0.6	0.04 ± 0.003	3.8 ± 0.2	8.4 ± 0.5	2.5 ± 0.4	85 ± 10	2.5 ± 0.3	1.2 ± 0.1	1.6 ± 0.3	2.5 ± 0.3	0.7 ± 0.1
WT IRI	B12	8.1 ± 0.8	0.02 ± 0.004	3.0 ± 0.3	6.9 ± 0.5	6.9 ± 0.3	19 ± 13	0.7 ± 0.4	1.5 ± 0.2	0.9 ± 0.3	0.7 ± 0.4	0.9 ± 0.1
Elmo1$^{H/H}$ IRI	B12	6.9 ± 1	0.02 ± 0.005	2.6 ± 0.3	6.4 ± 0.4	6.4 ± 0.3	26 ± 15	0.6 ± 0.5	1.7 ±	1.8 ± 0.4	0.6 ± 0.5	1.2 ± 0.1
p(ANOVA)												
Genotype (G)		0.9	0.04	0.9	0.9	< 0.0001	0.0032	0.1	0.3	0.04	0.1	0.08
Treatment (T)		0.0029	< 0.0001	0.0029	0.0007	< 0.0001	0.0225	0.0019	0.0094	0.9	0.0019	0.001
G x T interaction		0.09	0.17	0.9	0.2	0.0027	0.0140	0.09	0.6	0.5	0.09	< 0.0001

p-cystatin C: plasma cystatin C; u-ACR: urine albumin-to-creatinine ratio; eGFR: estimated glomerular filtration rate; Contra/BW: Contralateral kidney weight/Body weight; Affected/BW: Affected kidney weight/Body weight

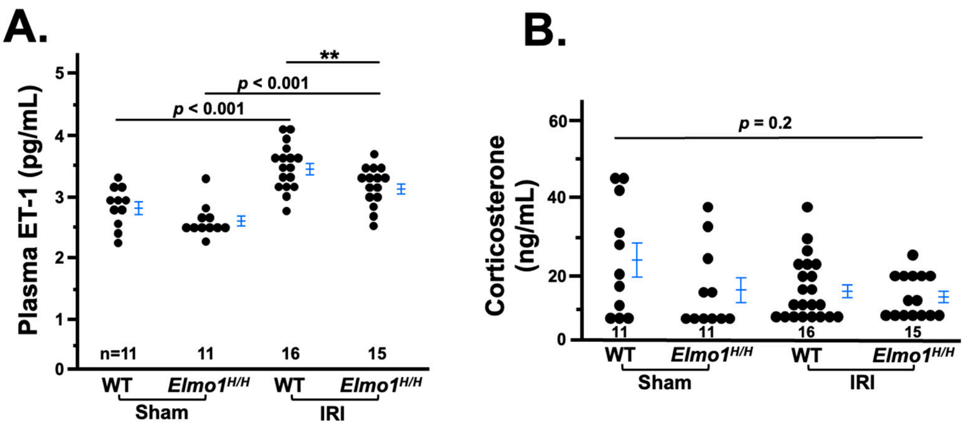

Supplemental Figure S1. Plasma endothelin-1 (ET-1) (**A**) and plasma corticosterone levels (**B**) across experimental groups. ** $p < 0.01$, p values smaller than 0.001 are listed as <0.001.

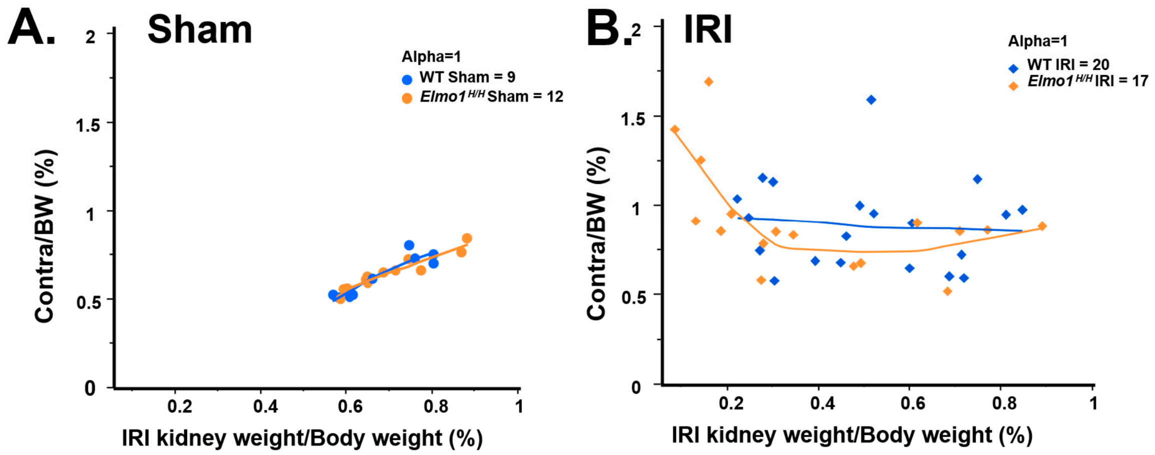

Supplemental Figure S2. Correlation between the affected (IRI kidney) and contralateral kidney (Contra) weights of each animal with sham (**A**) and IRI (**B**), presented by kernel smoothness, alpha=1. # of animals are indicated in the figures.

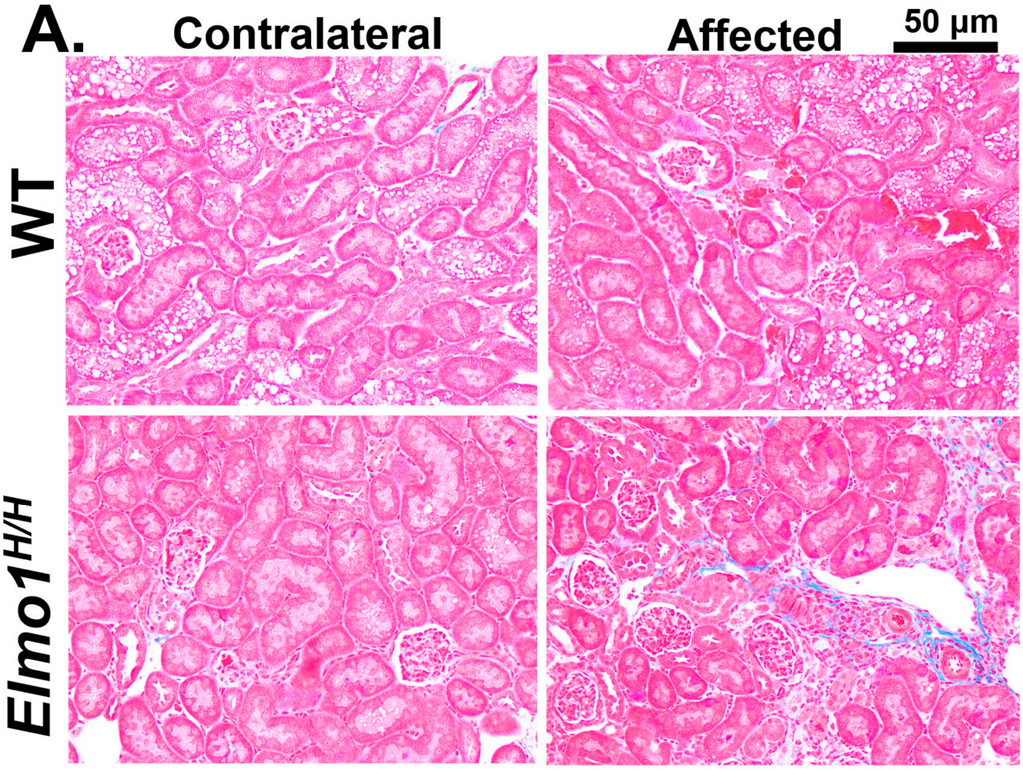

Supplemental Figure S3. **Kidney histological changes in wildtype and *Elmo1$^{H/H}$* male mice one month after IRI.** Masson's Trichrome staining showed low degree of interstitial tubular loss and fibrosis on *Elmo1$^{H/H}$* groups (200x).

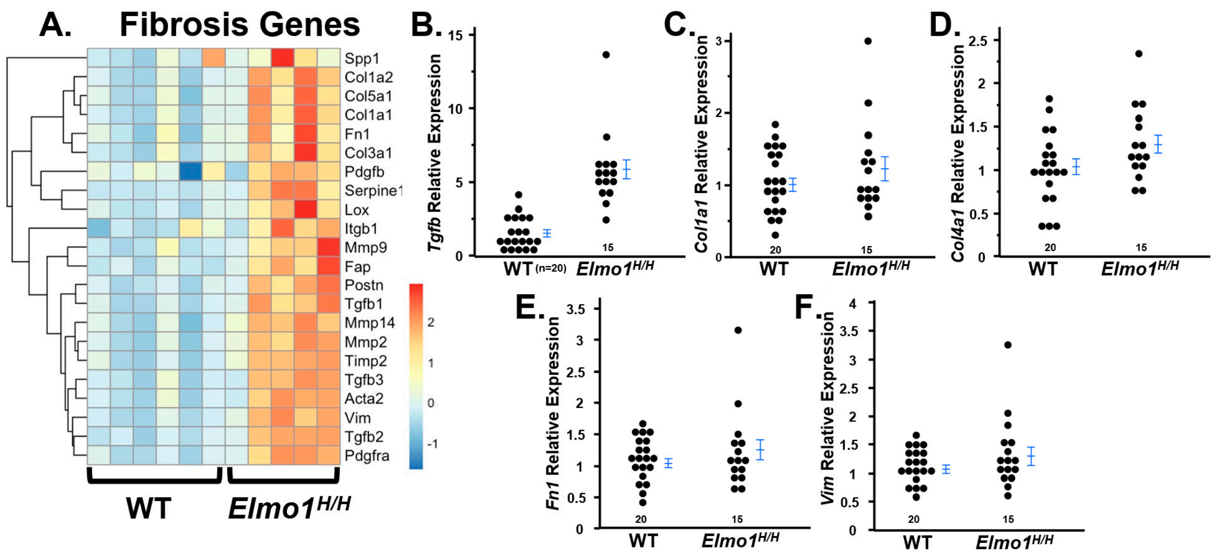

Supplemental Figure S4. Fibrosis gene expression shown by RNA-seq heatmap (**A**) in affected kidneys of WT (n=6) and *Elmo1*$^{H/H}$ (n=5) mice and by qRT-PCR (**B–F**) in contralateral kidneys ** $p < 0.01$, p values smaller than 0.001 are listed as <0.001.

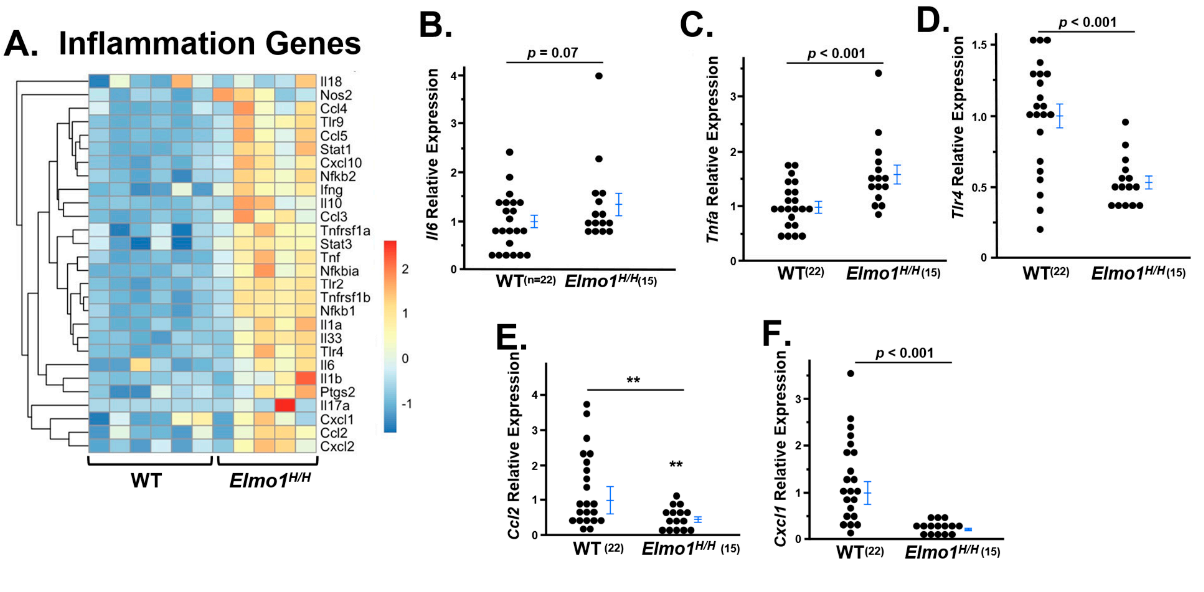

Supplemental Figure S5. Inflammatory gene expression shown by RNA-seq heatmap (**A**) in affected kidneys of WT (n=6) and *Elmo1*$^{H/H}$ (n=5) mice and by qRT-PCR (**B–F**) in contralateral kidneys. ** $p < 0.01$, p values smaller than 0.001 are listed as <0.001.

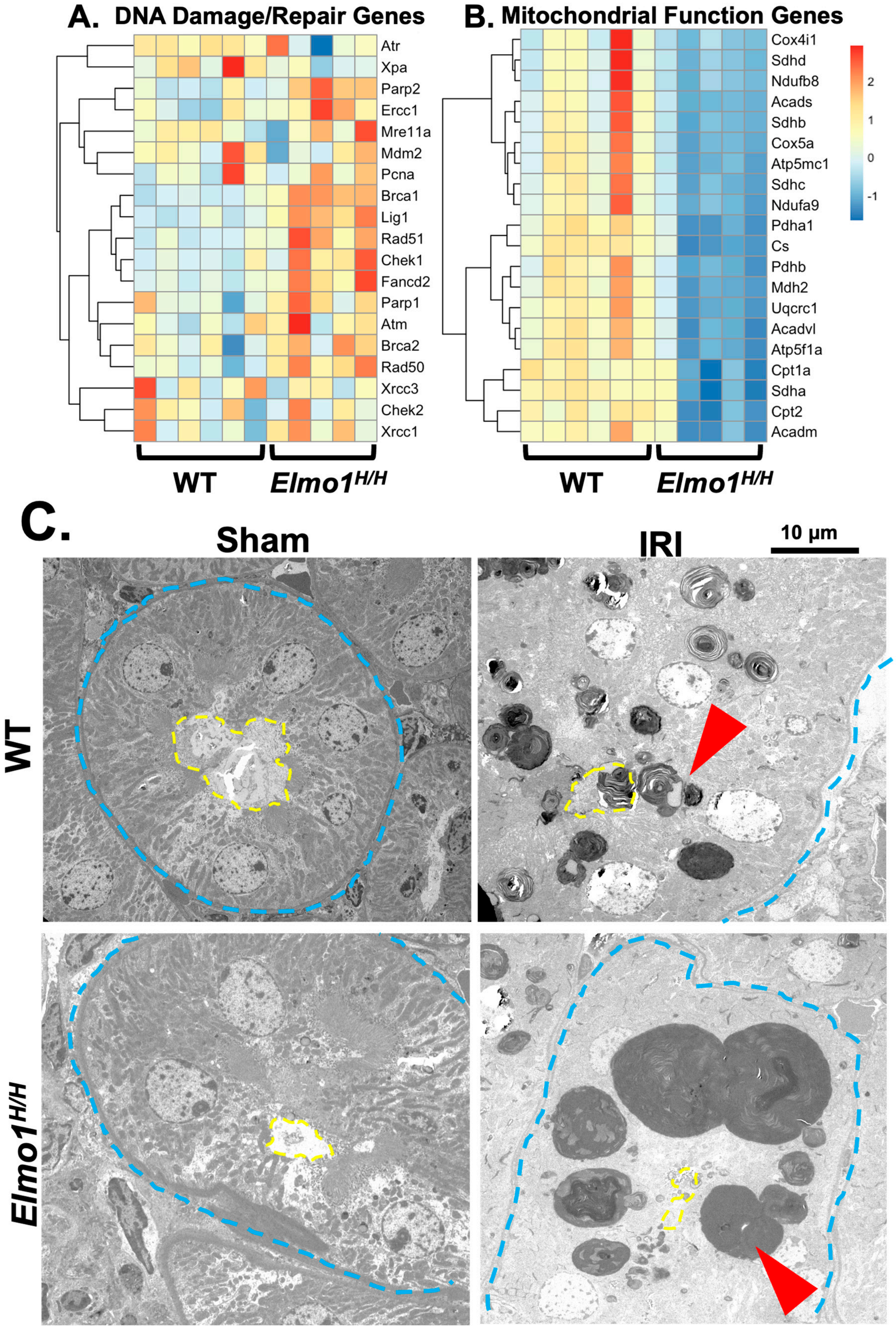
A. DNA Damage/Repair Genes
Atr
Xpa
Parp2
Ercc1
Mre11a
Mdm2
Pcna
Brca1
Lig1
Rad51
Chek1
Fancd2
Parp1
Atm
Brca2
Rad50
Xrcc3
Chek2
Xrcc1
WT
Elmo1H/H
B. Mitochondrial Function Genes
Cox4i1
Sdhd
Ndufb8
Acads
Sdhb
Cox5a
Atp5mc1
Sdhc
Ndufa9
Pdha1
Cs
Pdhb
Mdh2
Uqcrc1
Acadvl
Atp5f1a
Cpt1a
Sdha
Cpt2
Acadm
2
1
0
-1
WT
Elmo1H/H
C.
Sham
IRI
10 µm
WT
Elmo1H/H

Supplemental Figure S6. DNA damage and repair related genes and mitochondrial related gene expression shown by RNA-seq heatmap (**A-B**) in affected kidneys of WT (n=6) and *Elmo1*$^{H/H}$ (n=5) mice. (**C**) TEM of affected kidneys showing ceramides/lipids containing lysosomes (zebra bodies) in the proximal tubular cells (2000x, Red arrowhead), Blue dash line: border of a single proximal tubule. Yellow dash line: lumen of proximal tubule.

Reference

1. Dupont V, Berg AH, Yamashita M, et al. Impaired renal reserve contributes to preeclampsia via the kynurenine and soluble fms-like tyrosine kinase 1 pathway. *J Clin Invest*. Oct 17 2022;132(20)doi:10.1172/JCI158346
